# Public preparedness guidance for a severe influenza pandemic in different countries: a qualitative assessment and critical overview

**DOI:** 10.1371/currents.RRN1128

**Published:** 2010-04-15

**Authors:** Wladimir J. Alonso, Cynthia Schuck-Paim

**Affiliations:** ^*^Division of International Epidemiology and Population Studies (DIEPS); Fogarty International Center; National Institutes of Health and ^†^Scientific Consultant

## Abstract

During a severe influenza pandemic individuals and families can, by following well-directed and scientifically-based measures, not only benefit themselves but also play an effective role in reducing transmission rates and the burden on public services. Such guidelines should be provided as clearly and comprehensively as possible by official sources. Here we examine the official recommendations issued by 10 countries to prepare their citizens for a severe pandemic. We have found the presence of hazardous guidelines – as the advice to personally visit a health center at the earliest symptoms – and shortage of practical advices for home isolation, business preparation and treatment to be widespread. Our review shows that, while many positive recommendations were provided, the set of recommendations issued by most countries was not comprehensive enough for severe influenza scenarios. This is a situation that needs revision

## Introduction

The possibility of a human catastrophe produced by a novel, virulent and human-transmissible influenza strain introduced from wild and domestic animals is considered a plausible modern threat by renowned influenza experts [1] [2] [3] [4] [5] [6] [7]. As a consequence, in recent years the international community started to seriously prepare for a severe flu pandemic. Hence, the outbreak in Mexico of a new virus strain presenting all features of the feared pandemic (different age profile, origin from another species, high informed case fatality ratios - CFR) [8] and spreading in less than two months to most countries [10] rose the highest level of alert and concern among scientists, public health officials and governments worldwide [11] [12] . 

The development of events since then –mainly the adjustment to a milder CRF- has attenuated the perceived risk by the public and authorities [4] [12], but the prospect of a severe pandemic remains still plausible for future novel strains. With no vaccines for a long period and a limited availability of antivirals, medical care units and hospitals would be insufficient [11] [13] and basic public services could be disrupted in several levels. Still, in a severe pandemic crisis, individuals and families are expected to cope better and play a positive role in providing proper care to mild cases, reduce transmission rates and even make government-imposed mitigation measures viable if taking some critical steps in the right direction. Hence the importance of the public availability of comprehensive guidelines for pandemic preparedness that are well grounded on epidemiological knowledge and in an emergency planning framework. 

In the current study we critically examine the procedures put in place by official sources to prepare the public to that plausible worst-case situation, analyzing the appropriateness and effectiveness of the recommended measures to enable individual protection and help society. 

## Methodology

During May and June 2009 (a period when authorities still considered the H1N1 pandemic as a potentially serious threat) we examined the governmental websites from 10 countries chosen* ad libitum*, provided information was also available in English or Latin languages, and represented the official guidelines provided by the country. North America, South America, Europe, Oceania, Africa and Asia were represented in our sample, with 6 out of the 10 countries representing industrialized nations, and the sum of these 10 countries’ populations representing approximately 30% of the world population. 

National preparedness plans show a great variety in terms of their structure, level of detail and presence of annexes. It is important to highlight that our focus here is not on the National preparedness plans as a whole, but only on the preparedness recommendations issued directly to the public. 

We tabulated the existence of specific recommendations addressing the following aspects: how to prevent and reduce transmission of a respiratory disease, how to prepare for treating infected patients at home, how businesses should prepare, how to help the most vulnerable individuals of the neighborhood and how to prepare for home isolation (table 1). All information was recorded to enable independent consultation, but we will not disclosure the names of the countries here (unless when praise is possible) as it is not our intent to diminish the efforts of governmental agencies, but to constructively identify those aspects where improvement is possible.

It is important to note that in this field there are not unequivocal benchmarks or “golden standards” against which each guideline can be validated. Empirical data is not unequivocal -or even existing in some cases - and translation of scientific information and expertise knowledge into emergency planning guidelines is not automatic and without controversy. That is the reason why this study is mostly qualitative in nature rather than a quantitative analysis of this dataset – hence the discussion regarding the appropriateness of each measure should be interpreted mostly as the authors’ opinions on the subject. Finally, some of the arguments exposed here benefit from the current H1N1/2009 crisis, but we were careful in keeping a prudent distance from the evolving situation, as its mildness and the recent availability of vaccines correctly shifted recommendations to a “quasi-seasonal” scenario – certainly very different from the putatively severe one which originally inspired global concerns about an influenza pandemic.  

## Results and Discussion

As shown in table 1, only two recommendations are common to all 10 countries: washing hands and coughing etiquette. Other recommendations issued by more than half of the countries were not to touch eyes and mouth (7 countries), keep social distance when possible to avoid becoming ill (7 countries), isolate already infected patients at home (6 countries) and a phone number to contact (6 countries) to obtain information.

Table 1. Exemplifying some of the official preparedness recommendations (aggregated in major categories in the left-hand side column) provided by governmental sources. The number of countries (out of 10) adopting each recommendation is shown in the outermost column on the right.

**Table:** 

**Category**	**Specific recommendation**	**No of countries adopting it**
Reducing transmission to others	1. Isolation at home of infected individuals	8
	2. Treatment of patients at home (mild symptoms)	6
	3. Separate room	4
	4. Phone line to contact	6
	5. Call before visiting doctor	3
	6. Coughing etiquette	10
	7. Masks with handling advice	4
Prevention measures	1. Isolation at home when possible to avoid infection	7
	2. Not to touch eyes and mouth	7
	3. Washing hands	10
Treating patients at home	1. Antiviral without need to visit doctor	5
	2. Avoid dehydration	4
	3. How to treat fever	4
	4. Avoid aspirin for young individuals	4
Preparation for home isolation	1. Store water	2
	2. Store food	2
	3. Store medicines	2
	4. Store masks	1
	5. Store equipments for disruption	2
	6. Instructions on how to purify water	1
Business preparation	1. Tele-work, social distance, holidays	3
Helping those in need	1. Support for, e.g., the elderly, family of social workers	1

**Figure:**
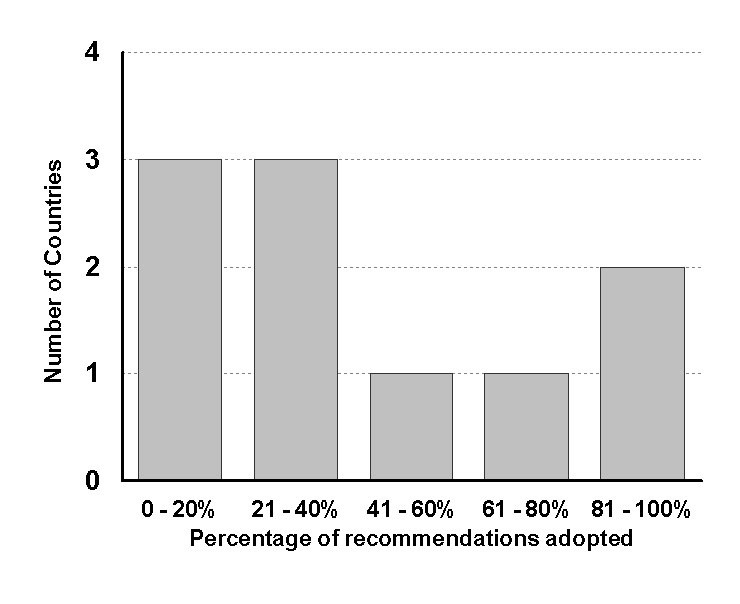

Figure 1. Overall percentage of recommendations adopted (out of the 22 listed in table 1) by the ten countries examined in this study. 

Figure 1 shows that only 2 countries issued more than 80% of all the recommendations listed in table 1. There were also only 2 countries which provided between 40% and 80% of these guidelines, with the remaining 6 countries providing less than 40% of the recommendations. In Figure 2 it becomes clear that most countries did not issue any of the recommendations regarding preparation for home isolation (8 countries do no mention any of the 6 actions listed in table 1, fig.2D), business preparation (not mentioned by 7 countries, fig.2E) and helping those in need (not mentioned by 9 countries, fig.2F). Specific actions needed for treating the ill at home are also not mentioned by 4 countries (fig.2C). Overall, the highest emphasis is placed on prevention measures (fig.2A).

**Figure:**
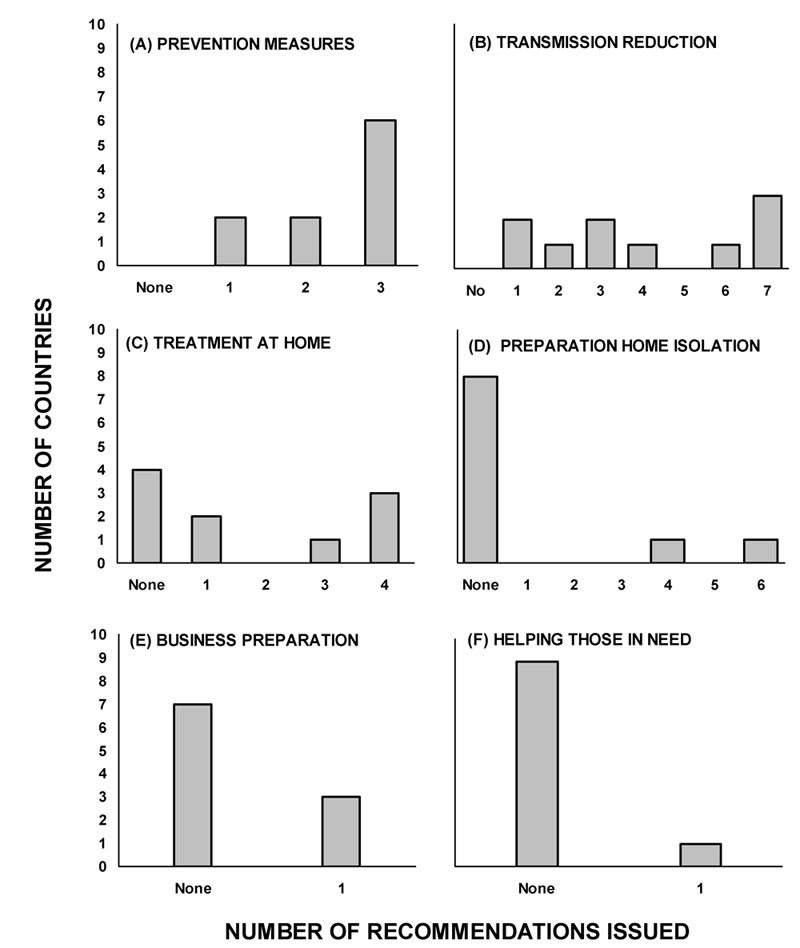

Figure 2.Number of recommendations (as based on those listed in table 1) adopted by the 10 countries examined in each of the major categories analyzed (A-F)

### Prevention Measures

Hand washing was the most ubiquitous recommendation given. While it is undeniable that it is an effective measure when treating patients with respiratory illnesses [14], there is no evidence of a high effectiveness for avoiding infection and transmission in everyday situations. By being covered by the dead cells of the external skin layer, hands are not suitable for virus replication – it is in the interaction of the hands with objects and with the mouth, nose and eyes [15] that viruses have an open door for reaching the living cells of the respiratory epithelium where they reproduce (Figure 3). Washing resets the hands from putative viruses’ load at that very moment, but recontamination can happen as soon as closing the tap, interacting with the knob when leaving the toilet (influenza viruses can remains viable up to two days in stainless steel and plastic surfaces) or simply handling bank notes [16]. In addition, the short survival times of influenza virus on human skin actually might imply that the most important factor regarding hands is not simply washing them, but avoid touching eyes and mouth [16], a recommendation that was issued by seven countries.

More crucial, however, is the observation raised by several studies that the aerosol route (Figure 3)  plays an important role in influenza transmission [16] [17]. In short, it implies that the simple physical proximity among individuals (without the need of physical contact) provides the conditions needed for efficient transmission. On the one hand, this means that it would be unfeasible and undesirable in our society to adopt the measures required to completely prevent transmission of seasonal influenza. But a severe pandemic (like the 1918’s, which in a few months led to more deaths than World War I caused in four years) calls for more stringent measures, which include the recommendation of avoidance of public places when possible, and the required complementary recommendations to make this possible. The understanding of the need of social isolation was, overall, widespread in our sample of countries, with 7 out of 10 countries mentioning isolation at home as a prevention measure. Nevertheless, the complementary information needed to make this recommendation viable to the population (that does not necessarily know how emergency measures can be effectively implemented) was only provided by 2 countries. We look at this in detail in the section “preparation for home isolation”.

**Figure:**
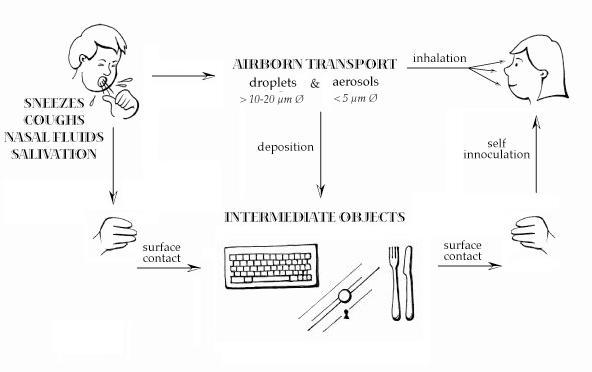

Figure 3. Main routes by which influenza viruses can be transmitted from an infected individual to a putatively susceptible one. Many aspects (like survival and transport of the virus depending on environmental conditions) can interfere in intermediate steps and their relative importance in ways that are still not completely quantified or understood by science (author: Diana Marques) .

### Reducing transmission to others

The epidemiological impact of reducing contact among susceptible and infected hosts as early as possible was translated by most countries in the recommendation that individuals should avoid close contact with others when suspicious of being infected. However, this relevant guideline looses critically its efficacy (and to a great extent its credibility) when contrasted with the recurrent and widespread advice (issued by 4 out of the 10 countries) to visit the nearest health center as one of the first measures that should be taken at the earliest influenza symptoms. 

In fact, unless there is a potential and immediate life threat, there is little justification to stimulate a putatively infected person to circulate in public places in a full-blown pandemic, with a very deadly strain of flu. Widespread telecommunications systems now enable the implementation of 24/7 hotlines (e.g., through telephone, internet), where the potentially infected person would receive self-triage guidelines, specific recommendations from health professionals and –if pertinent- an on-site visit of a doctor or ambulance. In fact, this initiative is also being adopted in some US states in order to “*both relieve hospitals and clinics of the enormous burden of screening thousands of flu patients, and, more important, provide the fastest access to antiviral medicine for people most at risk of developing life-threatening illness*” [18] [19]. 

Coughing etiquette was recommended by all countries. Advice on the use of masks was less frequent and sometimes confusing: for example, some countries recommended the use masks only prophylactically when travelling to countries with confirmed cases, when the country of origin already had cases itself. Masks can even produce adverse effects if not handled with caution. Yet, only 4 countries provided advice on the proper handling of masks.

### Treating patients at home

As mentioned previously, in most cases patients not only do not need to visit a doctor but also can be treated at home until full recovery. This is only possible if households are well informed on how to clearly identify the most severe cases (which, of course, have to be taken to hospitals immediately) and, on the other hand, how to treat those patients with mild influenza-like-illness. Otherwise, visits to medical units will take place at the earliest symptoms of the disease, increasing the likelihood of transmission to others if already infected or, conversely, increasing their own risk of infection if not infected yet. Still, only 4 countries issued instructions on how to avoid dehydration, treat fever and avoid giving aspirin to young individuals.

In face of a supposedly severe pandemic, half of the countries did not instruct their citizens on how to properly use antivirals. Instead, they just warned the population against its unsupervised use. Here too the strained context of a severe pandemic is being missed. Self-medication presents risks that can be greatly minimized through the consultation of qualified health professionals. But more realistically, one cannot expect that in a severe pandemic scenario overwhelmed doctors will be able to attend a great influx  of infected people in their clinics and issue prescriptions for medicines that patients should probably had to have already taken anyway. 

As mentioned, health professionals will not be able to tell much better than the patient herself if the symptoms represent a flu infection, as flu tests are time-consuming and expensive [20] and a waste of precious time for antiviral use [21]. Indeed, the delay in providing antivirals has been suggested as one of the causes for the high lethality of H1N1 in some countries [9]. Since logistic problems with antiviral distribution and availability are to be expected during a severe crisis, it becomes difficult to understand why antivirals (a highly time-sensitive medicine) could not be stocked by families and businesses that could afford it in inter-pandemic situations, when demand is low. In such cases, governmental stockpiles (which for most countries are not enough for the entire population) would have a lower demand to fulfill during a pandemic scenario. But, on the contrary, several countries included in their recommendations for a severe pandemic warnings against self-medication based on the risk that it would favor resistant strains. We do not agree with such judgment. First, because a proper treatment (under medical supervision or self-medication) should include all recommendations for preventing transmission, making the treated patient a dead end for the virus (in fact, the spread of resistance does not need to happen if the treated person remains quarantined –either at home or in the hospital- until full recovery). Second, because it is difficult to sustain on ethical grounds (and even harder to convince or enforce) that people should resign to provide a sub-optimal therapy to a severely ill family member based on a hypothetical reduction of the efficacy of the medication when used in the future by others.  

### Preparation for Home Isolation

Lack of basic goods usually trail catastrophes, aggravating the humanitarian crisis and opening the gate to surges of public arrest. There is nothing indicating that these circumstances should not be anticipated in case of a severe influenza pandemic, and authorities should be clear about such possibility and plan for it in advance. Families that can afford and want to embark on contingency plans during non-critical periods would be benefiting not only themselves by keeping distance from contagious exposure and shortage of goods, but also the society as a whole by lowering requirements in the most critical stages of a pandemic crisis (and -of course- by reducing the spread of the disease). 

A proper preparation should completely halt for a considerable period of time the direct social interactions through which people get various sorts of goods and services. Advices should include, for instance, information on how to store basic items (e.g., water, food, medicines, masks) [22] and provide tips in case of disruption of services (like how to obtain water in alternative ways). Because a fine balance must be found between the economic and social costs incurred by home isolation (hence the possibility and willingness of the population and businesses in supporting this recommendation) and how long a putatively serious disruption due to a severe influenza pandemic could last, there is no consensus for the time period a home supply of food and drinks should be planned for. The New Zealand and US official websites suggest a self-quarantine period (namely, a period of voluntary isolation at home) of one and two weeks, respectively [23] [24].

Such assessment does not seem to be shared by the majority of countries, which overly refrained from mentioning any practical advice on how to convert the very recommendation of home isolation (which, paradoxically, is suggested by most) into practical actions. It is worth mentioning that the official websites of the two countries mentioned above are excellent in terms of the coverage of the recommendations examined here, and deserve mentioning so others can use them as templates (or simply translate or link to them). In fact New Zealand even appropriately issued the influenza pandemic isolation recommendations within a broader and authoritative framework of catastrophe planning [25].  PandemicFlu is also an excellent source of information from the US [24], to where other agencies now link (reacting thus positively to constructive criticisms [26] made during the first stages of the H1N1 spread).

### Business preparation

In a mild crisis such as the on-going  H1N1/2009 (or for that matter, even seasonal influenza) simply asking symptomatic workers to stay at home is a measure that represents a good balance between the reduction of infection in the work place (although not 100% effective due to the presence of asymptomatic-infectious individuals) while ensuring the minimal possible disruption to businesses.  Still, this balance is radically different on the prospect of a severe pandemic. That is why pandemic preparedness measures should clearly inform businesses about more complex preparation. Having business well counseled on contingency plans would not only lower the transmission of the disease, but also make them repositories of logistic support for dealing with critical situations (operational business can stretch their capacity to help the community in many ways). However, only a minority of countries (30%, fig.2E) addressed this aspect.

### Helping those who are most in need

In critic situations it is natural to be overwhelmed with the need to provide care to family members and friends. As a result vulnerable individuals without many or strong social connections (like the elderly who live alone) might be left unattended to a perilous level. Also, personnel who work in essential services (who therefore will need to leave their dearest ones uncared while he/she is being exposed to a life-threatening situation) would be in a particularly delicate situation in which neighborhood solidarity can be of crucial value. The incorporation of advices like this is indicative of a high level of forthcoming thought and comprehensiveness in a pandemic plan. Unfortunately, we only found it addressed by 1 country.

## Conclusion

Due to influenza’s pervasive (and in many aspects still unknown) ways transmission [16] [27] [28], the uncertainty associated with its diagnosis, its frequent contagiousness without the presence of symptoms [29] and the uncertainty about the severity of pandemics as well as the pharmaceutical interventions that would be available, preparation for a severe pandemic is a complex matter even when analyzed only from an epidemiological perspective. From a political and public health context, we can also name, among others, the difficulties in investing political capital on a cause with a highly uncertain risk, as well as suggesting preparedness measures to a future health crisis when part of the population already lives in health straining conditions. However, as far as a severe crisis is believed to be plausible, the guidelines for such a scenario should be clearly available for citizens looking for that information [30].

In the current study, focused on the preparedness guidelines issued to the population by 10 countries, we found that, while many positive recommendations were provided, the set of recommendations issued by most countries was not comprehensive enough for severe influenza scenarios. Moreover, as we discussed along with the exposition of the results, some of the recommendations provided have proven to be inadequate to reduce transmission and enable an efficient allocation of limited resources to attend the most in need. Of course, during an on-going crisis the daily recommendations issued to the population should be tuned to the situation in progress at each moment. So, for instance, at the moment when this text is written, the relative mildness (in pandemic terms) of the H1N1/2009 would not justify the activation of home isolation recommendation for non-infected people. In fact, the mixed messages sent by the authorities may have lessened the willingness of the public to prepare for a future severe pandemic context. For instance, raising the pandemic alert to the highest possible level while the lethality of the strain has not proved specially severe has probably left the public with a perception that an influenza pandemic is not a reason for great concern, so it could be now more difficult to convince individuals to efficiently adopt the required preparedness measures in case of a severe pandemic.

Countries are expected to be prepared and work with great sense of self-sufficiency in light of a public health treat that, if materializing, is expected to occur globally. This includes even taking measures with a margin of risk higher than in normal situations (for example “*removing restrictions on the use of unapproved medical treatments and tests*” [4]).  In the same way, families should be also allowed to be prepared so as not to completely depend on outside help in such scenario. 

Beyond some direct interventions that can be enforced -like school and public transport closures [31] - the fact remains that, ultimately, transmission of a respiratory disease has multiple channels for finding its way in the social interactions among individuals. It is in this opportunistic dependency on numerous incidental individual behaviors where the transmission strength of the virus lies. Still, it is also where we can find opportunities for slowing down transmission in a very cost effective way, by providing clear, comprehensive and sound guidelines to the public. This is possible firstly because they are (given the scales involved) in a better position than countries to impose an isolation of their members, hence reducing the risk of exposure (at least during the peak of the crisis, when the possibilities of proper medical attention are more limited). Even putative government-imposed community mitigation measures– that were not within the scope of this study- are doubtlessly much more difficult to enforce if families are not prepared to capitalize on them (so, for instance, there is little use of  school closures if children are gathered in other settings due to a lack of contingency plans by their families). And second because, even when directly dealing with the disease, family units can provide proper care to mild cases (which even in the catastrophic scenario of 1918 corresponded to more than 95% of the cases in industrialized nations [6]), something that even the most developed countries would not be able to do in a crisis. In fact, one healthy adult can take care of several mild cases at home, provided he/she has the means and basic knowledge needed.

Finally, it is important to highlight that a set of recommendations for the population tuned to the magnitude of the event that is expect does not need to induce panic or irrational behaviors [26] (mainly if implemented as long-term preparedness measures during inter-pandemic periods). Instead, governments should start to consider citizens as valuable allies who, when well informed and guided, can be of effective help to manage and reduce the impact of a plausible severe crisis.

## Funding information

 Financial support for WJA was provided by intramural funds of the Fogarty International Center (from National Institutes of Health, USA)

## Competing interests

The authors have declared that no competing interests exist 
